# An open-label, randomized, phase 3 study of the efficacy and safety of antithrombin gamma in patients with sepsis-induced disseminated intravascular coagulation syndrome

**DOI:** 10.1186/s40560-018-0339-z

**Published:** 2018-11-16

**Authors:** Shigeatsu Endo, Ryutaro Shimazaki, the Antithrombin Gamma Study Group, Satoshi Gando, Shigeto Oda, Yasuhiro Ootomo, Masanao Miura, Shinji Ogura, Yutaka Eguchi, Joji Kotani, Norio Yamashita, Hiroyasu Ishikura, Yuichiro Sakamoto, Takeshi Takahashi, Yasushi Suzuki, Shigeki Kushimoto, Nobuya Kitamura, Naoyuki Matsuda, Takahiro Fujita, Mitsuhide Kitano, Junko Yamaguchi, Yoshikazu Yasuda, Hayato Takayama, Toshiharu Tanaka, Tetsuya Matsuoka, Tetsuhiro Takei, Tsuyoshi Hatada, Masahiro Tamashiro, Satoshi Fujimi, Osamu Nishida, Kazuhito Tamehiro, Junichi Maehara, Shinsuke Fujiwara, Hideo Wada

**Affiliations:** 1 Morioka Yuai Hospital, Nagai 12-10, Morioka, Iwate 020-0834 Japan; 2 R&D Division, Kyowa Hakko Kirin Co., Ltd., Tokyo, Japan

**Keywords:** Recombinant form of human AT, AT gamma, DIC, DIC recovery, Survival, JAAM DIC criteria, AT activity, Potelligent®

## Abstract

**Background:**

A recombinant form of antithrombin (AT), called AT gamma, is being developed as an alternative to AT derived from human plasma. To compare the efficacy and safety of AT gamma to plasma-derived AT (pAT), we conducted a randomized, open-label, multicenter trial in patients with sepsis-induced disseminated intravascular coagulation (DIC).

**Methods:**

Eligible patients, recruited at 30 clinical sites, had been diagnosed with sepsis-induced DIC (by the Japanese Association for Acute Medicine [JAAM] DIC criteria) and AT activity at 70% or below. Patients were randomized 1:1 to either 36 IU/kg/day AT gamma (*n* = 110) or 30 IU/kg/day pAT (*n* = 112), both administered intravenously for 5 days. The primary endpoint was recovery from DIC at day 6 or early study withdrawal. DIC recovery was defined as a DIC score of less than four. Secondary endpoints were DIC score, outcome on day 28, sequential organ failure assessment score, acute physiology and chronic health evaluation II score (APACHE II), and plasma AT activity. Adverse events and adverse drug reactions were recorded using MedDRA/J version 16.0.

**Results:**

Baseline patient demographics and clinical features were similar in the two treatment groups. On day 6 (or at withdrawal), DIC recovery had occurred in 62 of 110 (56.4%; 95% confidence interval, 46.6–65.8%) patients in the AT gamma group and 59 of 112 (52.7%; 95% confidence interval, 43.0–62.2%) patients in the pAT group. In both treatment groups, DIC recovery rate values tended to be higher when stratified by baseline AT activity rates. All changes in other secondary endpoints were similar in both treatment groups. Safety was also similar in the two treatment groups. Adverse events occurred in 89 of 108 (82.4%) patients in the AT gamma group and 99 of 113 (87.6%) patients in the pAT group.

**Conclusions:**

Safety and efficacy were similar for 36 IU/kg/day AT gamma and 30 IU/kg/day pAT. These results confirm that AT gamma is an excellent alternative to pAT for improving outcomes for patients with DIC.

**Trial registration:**

ClinicalTrials.gov identifier: NCT01384903; June 2011.

## Background

Antithrombin (AT), a major coagulation inhibitor in humans, is a single-chain glycoprotein with 432 amino acids and a molecular weight of approximately 58 kDa [1, 2]. AT binds and traps thrombin and other coagulation factors, including factors IX, X, XI, and XII [3, 4]. AT’s activity is enhanced 1000-fold by heparin [5].

Functional AT levels are significantly reduced in patients with congenital AT deficiency, and affected individuals are susceptible to clotting from a young age. Acquired AT deficiency can result from either decreased production or increased degradation of AT and is often associated with disseminated intravascular coagulation (DIC) [6], a life-threatening condition characterized by activated coagulation pathways, decreased anticoagulant activity, and altered activity of fibrinolytic pathways [6, 7]. In patients with DIC, numerous microthrombi form in the blood; however, patients may also experience excessive bleeding if clotting factors become depleted. DIC is typically secondary to other conditions such as sepsis and trauma [6].

Although the primary goal for treatment of patients with DIC is to address the underlying cause, various forms of AT have been developed that can be used to reduce the damage caused by DIC and improve survival. AT can also be used therapeutically to prevent clots in people with inherited AT deficiency, primarily during postoperative and perinatal time periods [8]. A large multinational randomized trial (KyberSept) found that AT treatment did not improve outcomes for patients with sepsis [9]. However, a reanalysis of the data focusing on the patients with DIC found a reduction in mortality in those patients treated with AT [10]. A systematic review of 32 trials confirmed this result [11]. A recent large, multicenter, retrospective study of 1784 patients diagnosed with DIC in Japan found that AT supplementation was associated with reduced mortality [12]. AT is widely used to treat DIC in Japan [8, 13]. The importance of AT and recommendations for its use are continually being evaluated as new data emerge.

A recombinant form of AT, called AT alfa (ATryn®), has been developed to provide a stable source of pathogen-free AT. Recombinant AT alfa, purified from the milk of goats that have been engineered to express human AT, is approved in the European Union and the USA for prevention of venous thromboembolism in patients with congenital AT deficiency during surgery [14]. However, the glycosylation profile of AT alfa differs from that of native AT, resulting in a dramatically reduced half-life [15]. To maintain adequate AT alfa levels, the drug must be administered by intravenous injection for 24 h.

AT gamma (KW-3357) is a new form of recombinant AT that has a sugar chain structure similar to that of native human AT. The drug was developed using Potelligent® technology, which relies on a host Chinese hamster ovary cell line that produces fucose-free recombinant proteins [16]. Native AT does not contain any fucose moieties, and because of this structural similarity, AT gamma has pharmacokinetic and biological activities similar to those of native AT.

AT gamma is being developed as an alternative to plasma-derived AT (pAT) products. Like pAT, AT gamma has both α and β isoforms (the α isoform is the dominant form, with an oligosaccharide occupying each of its four glycosylation sites, while the β isoform, representing 5–15% of the total AT found in human plasma [15], is a minor form that lacks glycosylation at one of these sites). For AT gamma, these isoforms are present in similar ratios to those seen for pAT (data on file, Kyowa Hakko Kirin Co., Ltd., Tokyo, Japan). Recombinant AT alfa also has both of these isoforms, and although the ratio has not been clearly defined, one report has suggested that the α isoform constitutes more than 80% [15]. Aside from the difference in the heterogeneity of the sugar chains, the main types of oligosaccharides (complex-type nonfucosylated N-linked oligosaccharides) in AT gamma and pAT are the same; however, AT gamma does not have any of the risks of infection that pAT has and can be stably produced without dependence on human plasma donors [15].

In a study in healthy male volunteers, AT gamma (72 IU/kg) was found to be bioequivalent to pAT (60 IU/kg) (data on file; Kyowa Hakko Kirin Co., Ltd., Tokyo, Japan). This multicenter, open-label, randomized, parallel-group, active-controlled, phase 3 study used doses of 36 IU/kg and 30 IU/kg for AT gamma and pAT, respectively. The objective was to evaluate the efficacy and safety of AT gamma in patients with septic DIC.

## Methods

### Patients

This study, conducted at 30 sites, enrolled patients aged ≥ 20 years with sepsis-induced DIC. Eligible patients met the American College of Chest Physicians/Society of Critical Care Medicine definitions for sepsis, had sepsis-induced DIC (calculated from DIC scores for four categories [systemic inflammatory response syndrome, platelets, prothrombin time (PT) ratio, and fibrinogen degradation product] of the Japanese Association for Acute Medicine (JAAM) DIC criteria [17]), and had AT activity ≤ 70%. The original protocol required patients to have AT activity from 50 to 70%, but the protocol was amended to include all patients with AT activity ≤ 70% so that the patient population would more accurately reflect the intended clinical use. In addition, the amendment allowed for use of thrombomodulin alfa (Recomodulin®). The following patients were excluded from the study: patients with drug allergies; patients with serious hepatic dysfunction; patients unlikely to survive long enough to provide efficacy and safety data; patients who were pregnant, breastfeeding, or who may have been pregnant; patients who had participated in another clinical study in the previous 4 months; patients who had previously received AT gamma; patients who had received prohibited concomitant medications or therapies between informed consent and enrollment; and patients judged to be ineligible by the investigator.

### Study design and treatments

**Fig. 1:**
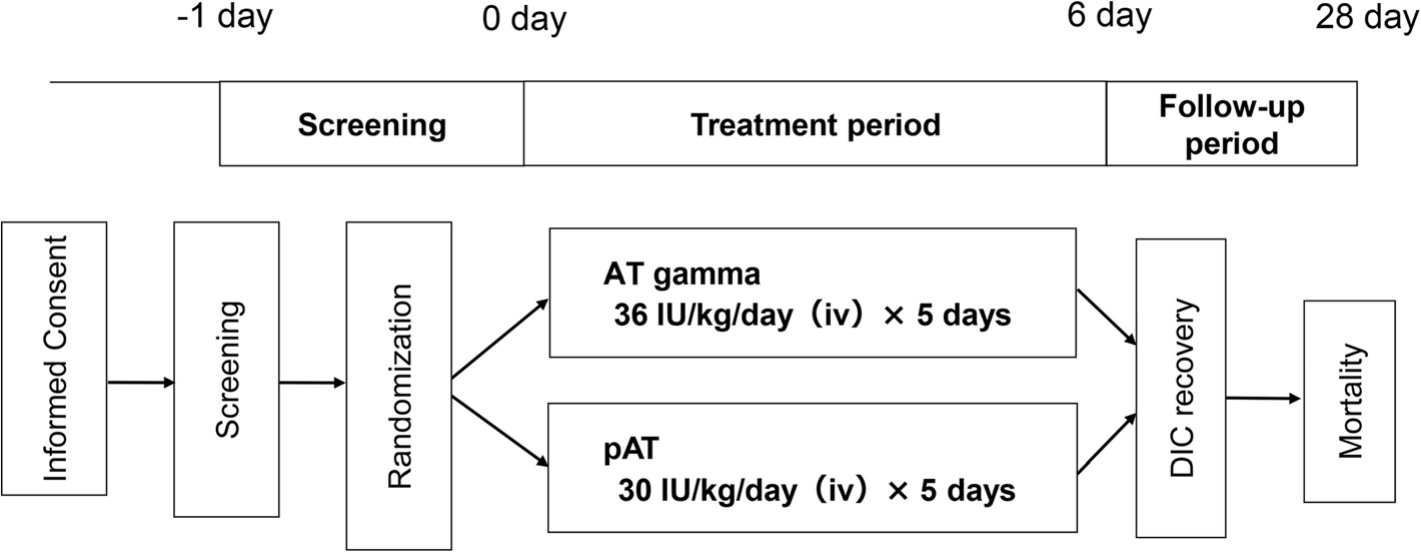
Study design. Eligible patients were randomized 1:1 to either 36 IU/kg/day AT gamma or 30 IU/kg/day pAT administered intravenously for 5 days. After 5 days of treatment, patients were examined on day 6 to assess DIC recovery and on day 28 to assess mortality. Heparins were also administered, except in patients for whom concomitant use of heparins could have increased the risk of bleeding

An enrollment center randomized patients 1:1 to the two groups using a permuted block design (Fig. 1). The study was conducted unblinded. The area under the curve for pAT was 1.2-fold higher than that for AT gamma in the bioequivalence study when using healthy adult Japanese men (data on file; Kyowa Hakko Kirin Co., Ltd., Tokyo, Japan). This was possibly due to the slightly smaller sialic acid content per AT gamma protein than that per pAT protein. Therefore, a 1.2-fold higher dose (72 IU/kg) of AT gamma was considered appropriate. Patients were treated with either 36 IU/kg/day AT gamma or 30 IU/kg/day pAT (human anti-thrombin III, Neuart® I.V., Japan Blood Products Organization) administered intravenously for 5 days. The daily dose was calculated based on the body weight measured at study enrollment. Heparins were also administered, except in patients for whom concomitant use of heparins could have increased the risk of bleeding. After 5 days of treatment, patients were examined on days 6 and 28. Patients withdrawing from the study before the examination on day 6 were examined promptly after withdrawal.

### Efficacy evaluation

The primary endpoint was the presence or absence of DIC recovery at either day 6 or at withdrawal. DIC recovery was defined as a DIC score of < 4 calculated according to the JAAM DIC criteria. The secondary endpoints were DIC score, survival of patients on day 28, sequential organ failure assessment (SOFA) score, acute physiology and chronic health evaluation (APACHE) II score, and plasma AT activity. AT activity was measured using the factor IIa method at a centralized laboratory (SRL Medisearch). Using this method, excess heparin was added to each AT sample to form an AT-heparin complex, which in turn binds excess thrombin. The AT-heparin complex inactivates the corresponding amount of excess thrombin, and residual thrombin activity was measured to determine the AT activity in the sample.

### Safety evaluation

The safety of AT gamma was also assessed in comparison to pAT. All adverse events (AEs) were coded using MedDRA/J, Version 16.0. Development of anti-AT gamma antibodies was assessed with an immunoassay. AT inhibition was assessed using a chromogenic synthetic substrate assay for AT activity.

### Sample size

The sample size for this study was set large enough that the 95% confidence interval for the DIC recovery rate for each treatment group could be estimated with an accuracy of ± 10%. The DIC recovery rate was assumed to be 50%, which maximizes the confidence interval width. The target sample size was 200 patients, with 100 patients per treatment group.

### Statistical methods

Categorical data were summarized as frequency and percentage, and continuous data were summarized with descriptive statistics (number of patients, mean, standard deviation [SD], minimum, median, and maximum). The primary evaluation time point was day 6 (or at withdrawal if before day 6). The DIC recovery rate and its 95% confidence interval (CI) were calculated for each treatment group. In addition, the (adjusted) DIC recovery rate and its 95% CI were calculated for each treatment group based on the Woolson–Bean method, stratified by the AT activity (< 50%, 50–70%) at study enrollment. For reference, DIC recovery at other time points was also analyzed in the same manner.

The secondary endpoints were analyzed at each time point, and the change from baseline was calculated for each. For outcome on day 28, survival rates were summarized for each treatment group. In addition, the (adjusted) survival rate was calculated for each treatment group based on the Woolson–Bean method stratified by the AT activity (< 50%, 50–70%) at study enrollment. Statistical analysis was performed using SAS 9.2.

## Results

### Patients

**Fig. 2:**
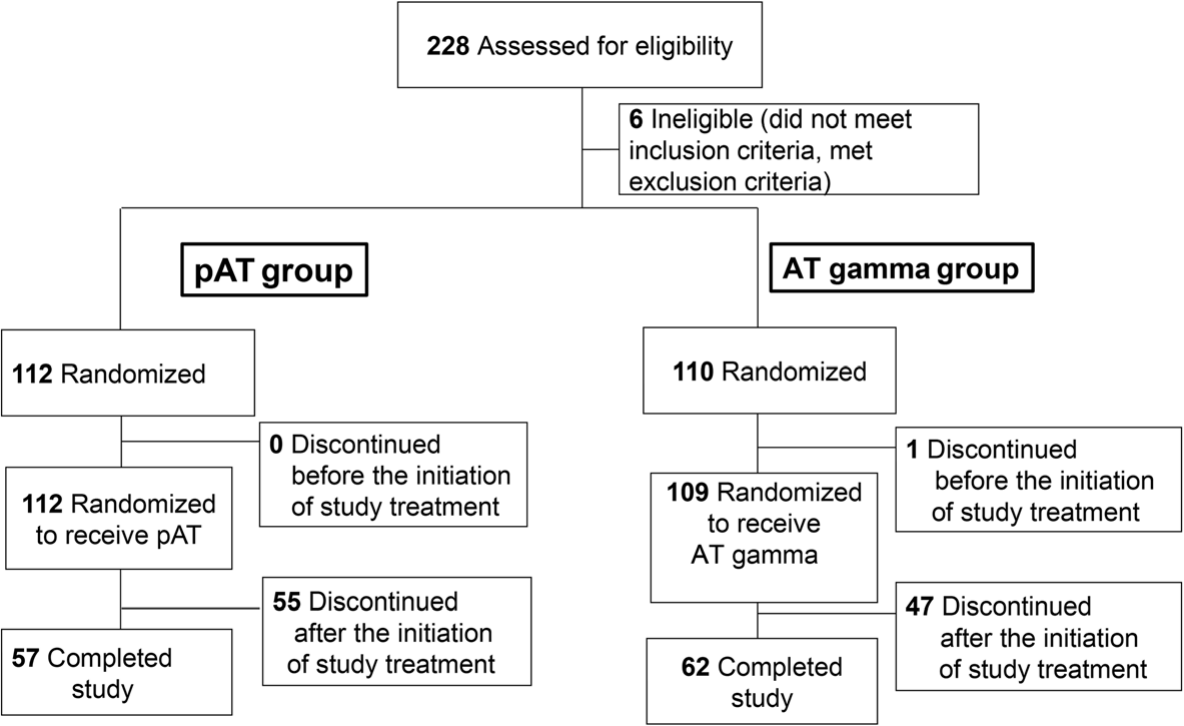
Patient disposition

In this study, 228 patients were enrolled, and 222 patients were randomized after exclusion of six patients judged to be ineligible for enrollment in the study (Fig. 2). The first informed consent was received in June 2011 and follow-up was completed for all patients by May 2013. Of the 112 patients assigned to the pAT group and 110 patients assigned to the AT gamma group, one patient (0.9%) in the AT gamma group withdrew from the study before the start of study treatment. Therefore, 221 patients received study treatment. One hundred and three patients (46.4%) withdrew from the study after randomization, including 48 (43.6%) in the AT gamma group and 55 (49.1%) in the pAT group.

Baseline demographic and clinical characteristics were similar in the two groups (Table 1). The mean (SD) age was 70.4 (15.2) in the AT gamma group and 71.0 (15.5) in the pAT group. The mean (SD) DIC score at enrollment was 5.6 (1.2) in the AT gamma group and 5.6 (1.4) in the pAT group. The mean (SD) SOFA scores at baseline were 9.1 (3.3) and 8.9 (3.7) in the AT gamma and pAT groups, respectively. The mean (SD) APACHE II scores at baseline were 18.2 (6.5) and 18.8 (6.8) in the AT gamma and pAT groups, respectively.

**Table 1:** Baseline patient characteristics

		pAT (*N* = 112)	AT gamma (*N* = 110)
Sex	Female	51 (45.5)	51 (46.4)
	Male	61 (54.5)	59 (53.6)
Age, years	Mean ± SD	71.0 ± 15.5	70.4 ± 15.2
	< 65	24 (21.4)	34 (30.9)
	65 to < 75	34 (30.4)	20 (18.2)
	75 to < 85	36 (32.1)	41 (37.3)
	≥ 85	18 (16.1)	15 (13.6)
Body weight, kg	Mean ± SD	54.3 ± 12.3	53.8 ± 14.0
	< 55	57 (50.9)	63 (57.3)
	≥ 55	55 (49.1)	47 (42.7)
Bleeding symptoms at baseline	Absent	90 (80.4)	93 (84.5)
	Present	22 (19.6)	17 (15.5)
DIC score at enrollment	Mean ± SD	5.6 ± 1.4	5.6 ± 1.2
	< 6	60 (53.6)	62 (56.4)
	≥ 6	52 (46.4)	47 (42.7)
SOFA score at enrollment	Mean ± SD	8.9 ± 3.7	9.1 ± 3.3
	< 10	64 (57.1)	58 (52.7)
	≥ 10	47 (42.0)	50 (45.5)
APACHE II score at enrollment	Mean ± SD	18.8 ± 6.8	18.2 ± 6.5
	< 20	68 (60.7)	61 (55.5)
	≥ 20	41 (36.6)	47 (42.7)
AT activity (%) at enrollment (on-site measurement)	Mean ± SD	50.9 ± 12.3	51.5 ± 11.6
	< 50	43 (38.4)	41 (37.3)
	50 to ≤ 70	69 (61.6)	68 (61.8)
Anticoagulant therapy use	Gabexate mesylate	22 (19.6)	21 (19.1)
	Nafamostat mesylate	6 (5.4)	4 (3.6)
	Thrombomodulin	35 (31.3)	34 (30.9)
	Heparins	31 (27.7)	32 (29.1)
Replacement therapy use	Fresh frozen plasma	24 (21.4)	10 (9.1)
	Platelet concentrate	26 (23.2)	22 (20.0)
Any anticoagulant use		83 (74.1)	76 (69.1)

*n* (%), *APACHE* acute physiology and chronic health evaluation, *AT* antithrombin, *DIC* disseminated intravascular coagulation, *pAT* plasma-derived antithrombin, *SOFA* sequential organ failure assessment

The protocol amendment on AT activity at enrollment did not result in any differences between the groups (Table 1). Mean AT activity was 51.5% in the AT gamma group and 50.9% in the pAT group. At enrollment, AT activity was less than 50% in 41 (37.3%) patients in the AT gamma group and 43 (38.4%) patients in the pAT group. AT activity was between 50 and 70% in 68 patients (61.8%) in the AT gamma group and 69 patients (61.6%) in the pAT group.

Anticoagulation therapy use was similar in the two groups (Table 1). Thrombomodulin was used by 30.9% in the AT gamma group and 31.3% of patients in the pAT group. Heparins were used by 29.1% in the AT gamma group and 27.7% of patients in the pAT group.

### Primary endpoint analysis

The primary efficacy analysis was conducted in 222 patients in the intention-to-treat population (110 patients in the AT gamma group and 112 patients in the pAT group). The results are provided in detail in Table 2. DIC recovery was observed in 62 of 110 (56.4%, 95% CI 46.6–65.8%) patients in the AT gamma group and 59 of 112 (52.7%, 95% CI 43.0–62.2%) patients in the pAT group. For patients with AT activity below 50% at baseline, the DIC recovery rate was 46.3% (19 of 41) in the AT gamma group and 46.5% (20 of 43) in the pAT group. For patients with AT activity between 50 and 70% at baseline, the DIC recovery rate was 61.8% (42 of 68) in the AT gamma group and 56.5% (39 of 69) in the pAT group.

**Table 2:** Primary endpoint: presence or absence of DIC recovery on day 6 (or at withdrawal)

AT activity (%)	DIC recovery	
	pAT	AT gamma
	% (*n/N*)	% (*n*/*N*)
< 50	46.5 (20/43)	46.3 (19/41)
50–70	56.5 (39/69)	61.8 (42/68)
Total [95% CI]	52.7 (59/112) [43.0–62.2]	56.4 (62/110) [46.6–65.8]
Adjusted^a^ [95% CI]	52.7 [43.6–61.9]	55.9 [46.7–65.1]

*AT* antithrombin, *CI* confidence interval, *DIC* disseminated intravascular coagulation, *pAT* plasma-derived antithrombin

^a^The protocol was amended to include all patients with AT activity ≤ 70% so that the patient population would more accurately reflect the intended clinical use. The (adjusted) DIC recovery rate and its 95% CI were calculated for each treatment group after stratification (using the Woolson–Bean method) by the AT activity (< 50%, 50–70%) at study enrollment

### Secondary endpoint analysis

**Fig. 3:**
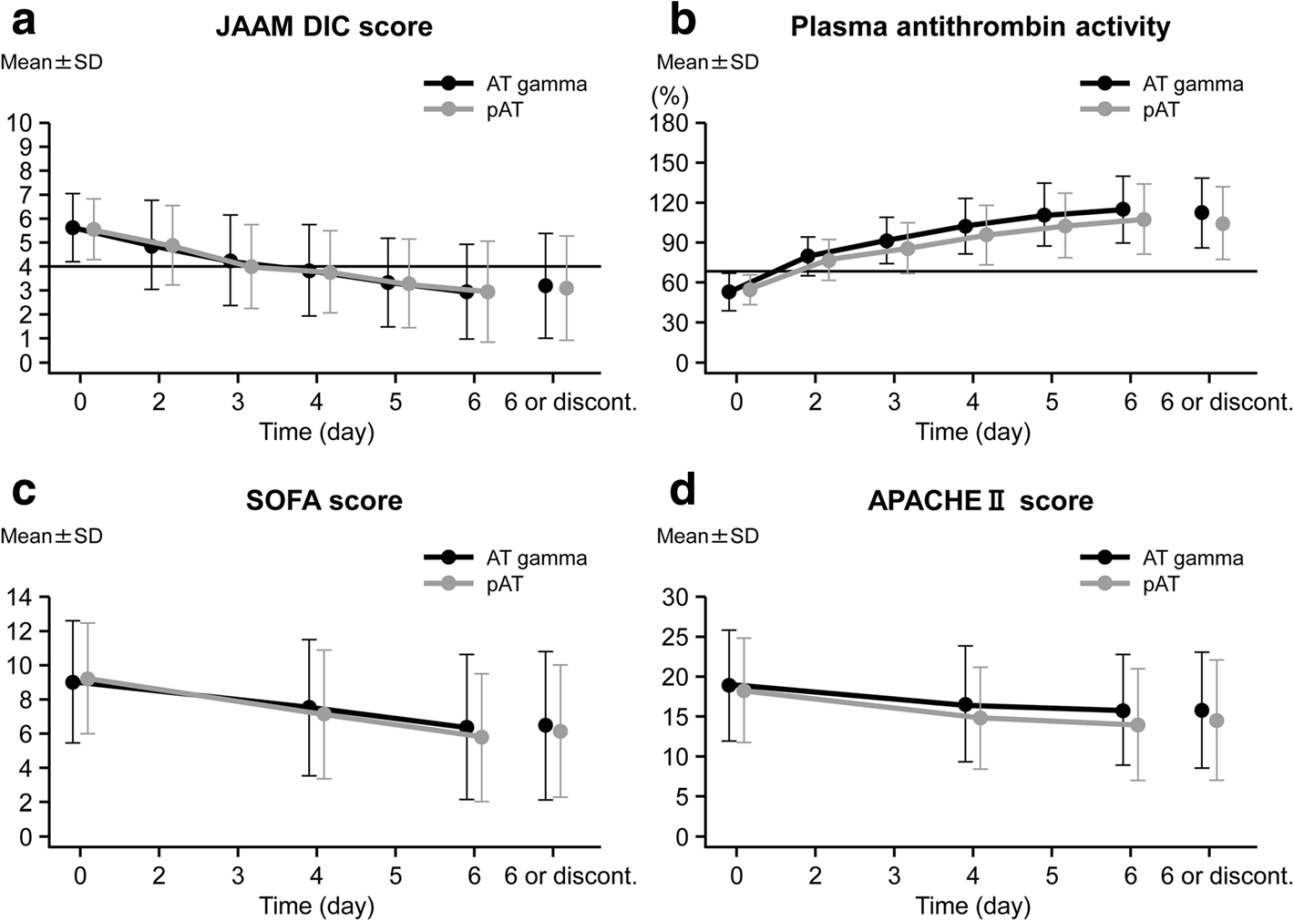
Changes in secondary endpoints during the study. The **a** DIC score (calculated from DIC scores for four categories [systemic inflammatory response syndrome, platelets, PT ratio, and FDP] of the JAAM DIC criteria), **b** plasma AT activity, **c** SOFA score, and **d** APACHE II score were assessed during the treatment and follow-up periods. The graphs show the mean values at each time point for the AT gamma (black) and pAT (gray) groups. The error bars indicate the standard deviation at each time point. APACHE, acute physiology and chronic health evaluation; AT, antithrombin; DIC, disseminated intravascular coagulation; discont., discontinuation or withdrawal; JAAM, Japanese Association for Acute Medicine; pAT, plasma-derived antithrombin; SOFA, sequential organ failure assessment

All improvements in secondary endpoints with AT gamma were comparable to those seen with pAT (Fig. 3). The change in the DIC score on day 6 (or at withdrawal) was − 2.4 ± 2.2 in the AT gamma group and − 2.4 ± 2.3 in the pAT group. The survival rate on day 28 was 87.3% (95% CI 79.6–92.9%) in the AT gamma group and 77.7% (95% CI 68.8–85.0%) in the pAT group (Table 3). The change in SOFA score on day 6 (or at withdrawal) was − 3.1 ± 3.3 in the AT gamma group and − 2.6 ± 3.6 in the pAT group. The change in APACHE II score on day 6 (or at withdrawal) was − 3.5 ± 6.1 in the AT gamma group and − 2.8 ± 6.1 in the pAT group. The change in plasma AT activity on day 6 (or at withdrawal) was 49.9% ± 23.7% in the AT gamma group and 58.8% ± 24.9% in the pAT group. The data regarding the changes of coagulation markers such as platelet count (PLT), fibrin/fibrinogen degradation products (FDP), and prothrombin time (PT) were also similar in both treatment groups. The mean ± SD changes in PLT, FDP, and PT were 3.28 ± 8.26 × 10^4^/μL vs. 4.83 ± 9.86 × 10^4^/μL, − 138.65 ± 649.01 μg/mL vs. − 111.81 ± 536.59 μg/mL, and − 2.65 ± 5.94 s vs. − 2.08 ± 3.85 s, respectively, in the pAT and AT gamma groups.

**Table 3:** Secondary endpoint: 28-day survival rate

AT activity (%)	Survival	
	pAT	AT gamma
	% (*n*/*N*)	% (*n*/*N*)
< 50	76.7 (33/43)	85.4 (35/41)
50–70	78.3 (54/69)	89.7 (61/68)
Total [95% CI]	77.7 (87/112) [68.8–85.0]	87.3 (96/110) [79.6–92.9]
Adjusted ^a^ [95% CI]	77.7 [70.7–84.7]	88.1 [81.1–95.0]

*AT* antithrombin*, CI* confidence interval, *pAT* plasma-derived antithrombin

^a^The protocol was amended to include all patients with AT activity ≤ 70% so that the patient population would more accurately reflect the intended clinical use. The (adjusted) survival rate and its 95% CI were calculated for each treatment group after stratification using the Woolson–Bean method, stratified by the AT activity (< 50%, 50–70%) at study enrollment

### Safety

The safety analysis set consisted of 221 patients (108 patients in the AT gamma group and 113 patients in the pAT group), after exclusion of one patient in the AT gamma group who withdrew before study treatment. There were no marked differences in the incidences of adverse events (AEs) and adverse drug reactions (ADRs) (Table 4). AEs occurred in 89 (82.4%) of 108 patients in the AT gamma group and 99 (87.6%) of 113 patients in the pAT group. ADRs occurred in 24 patients (22.2%) in the AT gamma group and 16 patients (14.2%) in the pAT group. The most common AE was erythema in both the AT gamma and pAT groups, occurring in 18 patients (16.7%) and 12 patients (10.6%), respectively (Table 5).

**Table 4:** Adverse events and adverse drug reactions

	pAT	AT gamma
	*N* = 113	*N* = 108
AEs, *n* (%)	99 (87.6)	89 (82.4)
AEs that led to death	22 (19.5)	10 (9.3)
Other serious AEs	7 (6.2)	14 (13.0)
ADRs, *n* (%)	16 (14.2)	24 (22.2)
ADRs that led to death	0 (0)	2 (1.9)
Other serious ADRs	1 (0.9)	3 (2.8)

*ADR* adverse drug reaction, *AE* adverse event, *pAT* plasma-derived antithrombin

**Table 5:** Adverse events occurring in at least 5% of patients in either treatment group

	pAT (*N* = 113)		AT gamma (*N* = 108)	
	Number of patients (%)	Number of events	Number of patients (%)	Number of events
Erythema	12 (10.6)	14	18 (16.7)	19
Diarrhea	6 (5.3)	6	17 (15.7)	18
Decubitus ulcer	10 (8.8)	12	12 (11.1)	13
Pneumonia	6 (5.3)	6	10 (9.3)	10
Vomiting	7 (6.2)	8	9 (8.3)	11
Constipation	4 (3.5)	5	9 (8.3)	9
Insomnia	10 (8.8)	10	9 (8.3)	9
Anemia	8 (7.1)	9	7 (6.5)	7
Sepsis	8 (7.1)	8	6 (5.6)	6
Hypokalemia	9 (8.0)	9	5 (4.6)	7
Hypernatremia	6 (5.3)	6	4 (3.7)	4
Pleural effusion	8 (7.1)	8	4 (3.7)	4
Nausea	7 (6.2)	7	3 (2.8)	4
Hepatic function abnormal	6 (5.3)	6	3 (2.8)	3
Blood bilirubin increased	6 (5.3)	6	3 (2.8)	3
Delirium	6 (5.3)	6	3 (2.8)	3
Skin exfoliation	11 (9.7)	17	3 (2.8)	3

*pAT* plasma-derived antithrombin

AEs that led to death occurred in 10 (9.3%) of the 108 patients in the AT gamma group and 22 (19.5%) of the 113 patients in the pAT group (Table 4). Of these, events that occurred in two patients (1.9%) in the AT gamma group were classified as ADRs. The most common AE that led to death was sepsis, occurring in four patients (3.7%) in the AT gamma group and seven patients (6.2%) in the pAT group. In addition, other serious AEs occurred in 14 (13.0%) of the 108 patients in the AT gamma group and seven (6.2%) of the 113 patients in the pAT group. Of these, events for which a causal relationship to the investigational product was not ruled out occurred in three patients (2.8%) in the AT gamma group (i.e., gastrointestinal hemorrhage, cerebral infarction, and hemorrhagic transformation stroke) and one patient (0.9%) in the pAT group (i.e., hemothorax). In the AT gamma group, there were 27 episodes of bleeding, and in the pAT group, there were 32. Apart from these, there were no other significant AEs.

In both the AT gamma and pAT groups, there were some patients whose laboratory values fluctuated during the study period or changed from baseline at the end-of-study examination. C-reactive protein levels, although they remained high, tended to decrease over time in both groups after the start of study treatment. However, for other test parameters, no consistent trend was observed. Transient changes in vital signs were observed in some patients in both groups, but no consistent trend was observed. All patients tested negative for antibodies to AT gamma at all time points.

## Discussion

This multicenter, open-label, randomized, parallel-group study investigated the efficacy and safety of AT gamma in patients with sepsis-induced DIC. All efficacy endpoints were similar in patients receiving 36 IU/kg/day AT gamma and patients receiving 30 IU/kg/day pAT. The protocol for this study was amended to include patients being treated with thrombomodulin and patients with AT activity below 50%. This change did not affect the analysis of the primary endpoint because there was no difference in AT activity or thrombomodulin use between the two groups. The rate of heparin use was also similar in the two groups.

With the inclusion of patients with AT activity below 50%, we were able to perform an analysis of the primary endpoint stratified by AT activity at enrollment (< 50% or 50–70%). The rate of DIC recovery was similar in the two treatment groups for patients with less than 50% AT activity at baseline (46.3% vs. 46.5% for AT gamma and pAT, respectively). The DIC recovery rate was 61.8% in patients in the 50–70% AT activity group receiving AT gamma and 56.5% in patients receiving pAT, but no test of the significance of this difference was specified in the analysis plan. In patients being treated with thrombomodulin, the DIC recovery rate was 64.7% in the AT gamma group and 40.0% in the pAT group. Survival rates on day 28 were 87.3% vs. 77.7% for the AT gamma group and pAT group, respectively, regardless of AT activity at enrollment. The survival rate on day 28 was similar in patients not using thrombomodulin, but in patients using thrombomodulin, the rates were 94.1% vs. 71.4% in the AT gamma and pAT groups, respectively.

The safety of AT gamma was comparable to that of pAT in this study. The lists of AEs occurring in at least 5% of patients were similar in the AT gamma and pAT groups. Common AEs included erythema, diarrhea, and decubitus ulcer. The number of bleeding events was similar in the two groups, and bleeding was infrequent even with concomitant use of thrombomodulin and heparin. In a separate clinical study, we previously found that AT gamma is also effective for patients with DIC secondary to blood disorders or malignant tumors (unpublished observation, S. Endo, Morioka Yuai Hospital, Morioka, Japan).

AT gamma is a recombinant AT preparation produced in Chinese hamster ovary host cells. This novel preparation of AT has the same amino acid sequence and the same main type of oligosaccharides (complex-type nonfucosylated N-linked oligosaccharides) as native human AT and, therefore, also has similar biological activity. Because the existing AT preparation is derived from human blood, safety measures against infectious diseases, such as virus inactivation and removal, must be performed during the manufacturing process; however, the risk of transmission of an infectious agent cannot be completely excluded. Furthermore, plasma-derived products are dependent on blood donations, and the blood donation population is not guaranteed. Because AT gamma is a genetically modified preparation, there is no risk of infection and the supply can be stably maintained.

Although recombinant AT alfa has been approved for use in patients with congenital AT deficiency, its half-life is markedly shorter than the half-life of native AT, and it must be administered continuously for 24 h. In contrast, AT gamma has been developed with a similar glycosylation profile to native AT, and its longer half-life than AT alfa allows for a short, 1-h infusion. We expect that the 1-h infusion will be more convenient in the hospital.

Our study has some limitations that should also be considered. First, the study was conducted open-label and was not designed to statistically compare the two treatment groups. Second, the study enrolled only patients with DIC with underlying infectious disease. Patients with other causes of DIC might respond differently to AT gamma. Third, long-term outcomes beyond 28 days were not assessed. Lastly, approximately half of the patients in the study were being treated with other anticoagulation therapies during the study.

## Conclusions

The results of this study demonstrate that 36 IU/kg/day of AT gamma is as efficacious and safe as 30 IU/kg/day of pAT. Thus, this study establishes AT gamma as an alternative to pAT for addressing this critical clinical need.

## Abbreviations

ADR: Adverse drug reaction
AE: Adverse event
APACHE: Acute physiology and chronic health evaluation
AT: Antithrombin
CI: Confidence interval
DIC: Disseminated intravascular coagulation (syndrome)
FDP: Fibrin/fibrinogen degradation products
pAT: Plasma-derived antithrombin
PLT: Platelet count
PT: Prothrombin time
SOFA: Sequential organ failure assessment
